# Response to Letter to the Editor From Josef Finsterer: “Explaining Long COVID: A Pioneer Cross-Sectional Study Supporting the Endocrine Hypothesis”

**DOI:** 10.1210/jendso/bvae087

**Published:** 2024-04-26

**Authors:** Taieb Ach, Nassim Ben Haj Slama, Asma Gorchane, Asma Ben Abdelkrim, Meriem Garma, Nadia Ben Lasfar, Foued Bellazreg, Widéd Debbabi, Wissem Hachfi, Molka Chadli Chaieb, Monia Zaouali, Amel Letaief, Koussay Ach

**Affiliations:** Department of Endocrinology, University Hospital of Farhat Hached Sousse, Sousse 4000, Tunisia; University of Sousse, Faculty of Medicine of Sousse, Sousse 4000, Tunisia; Laboratory of Exercise Physiology and Pathophysiology; L.R.19ES09; Department of Endocrinology, University Hospital of Farhat Hached Sousse, Sousse 4000, Tunisia; University of Sousse, Faculty of Medicine of Sousse, Sousse 4000, Tunisia; Department of Endocrinology, University Hospital of Farhat Hached Sousse, Sousse 4000, Tunisia; University of Sousse, Faculty of Medicine of Sousse, Sousse 4000, Tunisia; Department of Endocrinology, University Hospital of Farhat Hached Sousse, Sousse 4000, Tunisia; University of Sousse, Faculty of Medicine of Sousse, Sousse 4000, Tunisia; University of Sousse, Faculty of Medicine of Sousse, Sousse 4000, Tunisia; Infectious Diseases Department, Farhat Hached Hospital, Sousse 4000, Tunisia; University of Sousse, Faculty of Medicine of Sousse, Sousse 4000, Tunisia; Infectious Diseases Department, Farhat Hached Hospital, Sousse 4000, Tunisia; University of Sousse, Faculty of Medicine of Sousse, Sousse 4000, Tunisia; Infectious Diseases Department, Farhat Hached Hospital, Sousse 4000, Tunisia; University of Sousse, Faculty of Medicine of Sousse, Sousse 4000, Tunisia; Department of Endocrinology, University Hospital of Ibn El Jazzar, Kairouan 4071, Tunisia; University of Sousse, Faculty of Medicine of Sousse, Sousse 4000, Tunisia; Infectious Diseases Department, Farhat Hached Hospital, Sousse 4000, Tunisia; Department of Endocrinology, University Hospital of Farhat Hached Sousse, Sousse 4000, Tunisia; University of Sousse, Faculty of Medicine of Sousse, Sousse 4000, Tunisia; Department of Endocrinology, University Hospital of Farhat Hached Sousse, Sousse 4000, Tunisia; University of Sousse, Faculty of Medicine of Sousse, Sousse 4000, Tunisia; Laboratory of Exercise Physiology and Pathophysiology; L.R.19ES09; University of Sousse, Faculty of Medicine of Sousse, Sousse 4000, Tunisia; Infectious Diseases Department, Farhat Hached Hospital, Sousse 4000, Tunisia; Department of Endocrinology, University Hospital of Farhat Hached Sousse, Sousse 4000, Tunisia; University of Sousse, Faculty of Medicine of Sousse, Sousse 4000, Tunisia

**Keywords:** COVID-19, SARS-CoV-2, hypopituitarism, central adrenal insufficiency, GH deficiency, insulin tolerance test

Dear Editor,

We have read Dr Finsterer’s letter with great interest [1]. We appreciate the insightful commentary provided regarding our recent article investigating the endocrine implications in long-COVID patients [2].

The author highlighted the importance of considering chronic stress and its potential impact on the hypothalamic-pituitary-adrenal axis in long-COVID patients. In a recent meta-analysis, the pooled prevalence of depression and anxiety among patients with long-COVID syndrome was estimated to be 23% [3]. In this context, it is recognized that chronic stress would exacerbate the cortisol response, potentially leading to a pseudo-Cushing state [4]. However, it is crucial to emphasize that our study primarily focused on evaluating corticotropic insufficiency. Consequently, the inclusion of stress assessments might have resulted in a diminished number of patients diagnosed with corticotropic insufficiency because of the masking effect of stress-induced cortisol elevation, instead of a higher prevalence of corticotropic insufficiency.

The concern regarding the potential central nervous system involvement in causing pituitary insufficiency in the acute stage of SARS-CoV-2 infection is indeed valid. Determining the actual occurrence rate of hypophysitis following COVID-19 proves to be challenging [4]. There are only some cases that were reported, with 1 case of infundibuloneuro-hypophysitis [5]. However, our study focused on evaluating endocrine function during the postinfection period. Pituitary hormone determination and magnetic resonance imaging scans in the acute stage of SARS-CoV-2 infection were not within the scope of our study.

The author also highlighted a discrepancy in the exclusion criteria. All patients included in the study underwent thyroid assessment, with results values within normal limits. Regarding diabetes, patients were evaluated before the insulin tolerance test, and they exhibited normal glycemic parameters to achieve hypoglycemia. Although some patients had a history of certain conditions, these were well-controlled and did not present significant deviations from normal values.

For the final point, the alternative hypotheses proposed to explain long-COVID are indeed critical considerations in understanding the multifaceted nature of this syndrome [6]. Although our study primarily focused on exploring the endocrine hypothesis, we acknowledge the importance of addressing these alternative mechanisms comprehensively.

The multifaceted nature of the virus, with its ability to affect multiple tissues and organs through various mechanisms, underscores the complexity of long-COVID [7]. Although endocrine involvement may play a role in some aspects of long-COVID, it is evident that the syndrome encompasses a wide array of manifestations that cannot be solely attributed to endocrine dysfunction. Therefore, understanding long-COVID requires a comprehensive exploration of the interplay between viral pathophysiology, immune dysregulation, inflammatory processes, and other potential mechanisms, reflecting the intricate nature of this condition [8].

In summary, we appreciate the author's insightful comments on our study. Various hypotheses exploring these symptoms were raised; however, a closer examination of the residual symptoms experienced by these patients reveals that some closely resemble the symptoms associated with anterior pituitary deficiencies, particularly corticotroph or somatotroph. Consequently, the endocrine hypothesis involving anterior pituitary insufficiency was considered to explain long-COVID syndrome.

## Data Availability

No data were used for this manuscript.

## References

[1] Josef F . Letter to the editor from Josef Finsterer: “explaining long COVID: a pioneer cross-sectional study supporting the endocrine hypothesis”. J Endocr Soc. 2024. 10.1210/jendso/bvae086 PMC11089478 38741940

[2] Ach T, Ben Haj Slama N, Gorchane A, et al Explaining long COVID: a pioneer cross-sectional study supporting the endocrine hypothesis. J Endocr Soc. 2024;8(3):bvae003. 10.1210/jendso/bvae003 PMC10801829 38260089

[3] Seighali N, Abdollahi A, Shafiee A, et al The global prevalence of depression, anxiety, and sleep disorder among patients coping with post COVID-19 syndrome (long COVID): a systematic review and meta-analysis. BMC Psychiatry. 2024;24(1):105. 38321404 10.1186/s12888-023-05481-6 PMC10848453

[4] Taieb A, Nassim BHS, Asma G, Jabeur M, Ghada S, Asma BA. The growing understanding of the pituitary implication in the pathogenesis of long COVID-19 syndrome: a narrative review. Adv Respir Med. 2024;92(1):96‐109. 38392036 10.3390/arm92010013 PMC10886368

[5] Capatina C, Poiana C, Fleseriu M. Pituitary and SARS CoV-2: an unremitting conundrum. Best Pract. Res. Clin. Endocrinol Metab. 2023;37(4):101752. 36878774 10.1016/j.beem.2023.101752 PMC9969757

[6] Davis HE, McCorkell L, Vogel JM, Topol EJ. Long COVID: major findings, mechanisms and recommendations. Nat Rev Microbiol. 2023;21(3):133‐146. 36639608 10.1038/s41579-022-00846-2 PMC9839201

[7] Li MY, Li L, Zhang Y, Wang XS. Expression of the SARS-CoV-2 cell receptor gene ACE2 in a wide variety of human tissues. Infect Dis Poverty. 2020;9(1):45. 32345362 10.1186/s40249-020-00662-x PMC7186534

[8] Tziolos NR, Ioannou P, Baliou S, Kofteridis DP. Long COVID-19 pathophysiology: 133 what do we know so far?. Microorganisms. 2023;11(10):2458. 37894116 10.3390/microorganisms11102458 PMC10609046

